# Clinical features and outcomes of COVID-19 patients hospitalized for psychiatric disorders: a French multi-centered prospective observational study

**DOI:** 10.1017/S0033291721001537

**Published:** 2021-04-27

**Authors:** Daniela Dobre, Raymund Schwan, Claire Jansen, Thomas Schwitzer, Olivier Martin, Fabienne Ligier, Benjamin Rolland, Pierre Abdel Ahad, Delphine Capdevielle, Emmanuelle Corruble, Pascal Delamillieure, Sonia Dollfus, Dominique Drapier, Djamila Bennabi, Fabien Joubert, William Lecoeur, Catherine Massoubre, Antoine Pelissolo, Mathilde Roser, Christophe Schmitt, Noé Teboul, Clément Vansteene, Wanda Yekhlef, Antoine Yrondi, Radoine Haoui, Raphaël Gaillard, Marion Leboyer, Pierre Thomas, Philip Gorwood, Vincent Laprevote

**Affiliations:** 1 Centre Psychothérapique de Nancy, Laxou F-54520, France; 2 INSERM U1114, Fédération de Médecine Translationnelle de Strasbourg, Département de Psychiatrie, Centre Hospitalier Régional Universitaire de Strasbourg, Strasbourg F-67 000, France; 3 Faculté de Médecine, Université de Lorraine, F-54500 Vandoeuvre-lès-Nancy, France; 4 EA 4360 APEMAC, Université de Lorraine, F-54500 Vandoeuvre-lès-Nancy, France; 5 Service Universitaire d'Addictologie de Lyon (SUAL), CH Le Vinatier, Bron, France; 6 Services hospitalo-universitaires d'addictologie, Hospices Civils de Lyon, Lyon, France; 7 Université de Lyon, UCBL, Centre de recherche en neurosciences de Lyon (CRNL), INSERM U1028, CNRS UMR5292, PSYR2, Bron, France; 8 Pôle hospitalo-universitaire de psychiatrie adultes Paris 15ème, GHU Paris psychiatrie et neurosciences, site Sainte-Anne, Paris, France; 9 IGF, Univ. Montpellier, CNRS, INSERM, Montpellier, France; 10 University Department of Adult Psychiatry, CHU, Montpellier, France; 11 Université department of Adult Psychiatry, Hôpital La Colombière, CHU de Montpellier, France; 12 Service Hospitalo-Universitaire de Psychiatrie de Bicêtre, Hôpitaux Universitaires Paris-Saclay, Assistance Publique-Hôpitaux de Paris, Hôpital de Bicêtre, Le Kremlin Bicêtre F-94275, France; 13 CHU de Caen, Service de psychiatrie, Centre Esquirol, Caen F-14000, France; 14 Imagerie et Stratégies Thérapeutiques de la Schizophrénie (ISTS) EA 7466, Normandie Univ, GIP Cyceron, Caen F-14000, France; 15 UFR Santé, Normandie Univ, Caen F-14000, France; 16 Pôle Hospitalo-Universitaire de Psychiatrie Adulte, Centre Hospitalier Guillaume Régnier, Rennes F-35703, France; 17 EA 47 12 Comportement et Noyaux Gris Centraux, Université Rennes 1, Rennes F-35703, France; 18 Service de psychiatrie de l'adulte, CHRU de Besançon, F-25000 Besançon, France; 19 Centre expert dépression résistante FondaMental, F-25000 Besançon, France; 20 Département d'Information Médicale, CH Le Vinatier, Bron, France; 21 Établissement Public De Santé Alsace Nord, Brumath, France; 22 Service Universitaire de Psychiatrie, EA TAPE 7423, CHU de Saint-Etienne, Saint Etienne, France; 23 UPEC, Université Paris-Est, Faculté de médecine, Créteil F-94000, France; 24 AP-HP, DMU IMPACT, Hôpitaux universitaires Henri-Mondor, Service de Psychiatrie, Créteil F-94000, France; 25 INSERM U955, Laboratoire Neuro-Psychiatrie translationnelle, Créteil F-94000, France; 26 Département d'Information Médicale, Centre Hospitalier de Jury, Metz F-57073, France; 27 Clinique des Maladies Mentales et de l'Encéphale (CMME), Hôpital Sainte-Anne, 1 Rue Cabanis, 75014 Paris, France; 28 INSERM U894, Centre de Psychiatrie et Neurosciences (CPN), Université Paris Descartes, PRES Sorbonne Paris Cité, Paris, France; 29 Département Soins Somatiques-Préventions-Santé Publique, Pôle CRISTALES, EPS de Ville-Evrard, Neuilly sur Marne, France; 30 Service de Psychiatrie et de Psychologie Médicale, Centre Expert Dépression Résistante FondaMental, CHU de Toulouse, Hôpital Purpan, Toulouse, France; 31 ToNIC Toulouse NeuroImaging Center, Université de Toulouse, INSERM, UPS, Toulouse, France; 32 Pôle de Psychiatrie Générale Rive Gauche, Centre Hospitalier Gérard Marchant, F-31057 Toulouse, France; 33 Université de Paris, Paris, France; 34 Human Histopathology and Animal Models, Infection and Epidemiology Department, Institut Pasteur, Paris, France; 35 Univ. Lille, INSERM U1172, CHU Lille, Centre Lille Neuroscience & Cognition (PSY), F-59000 Lille, France; 36 CHU Lille, Pôle de Psychiatrie, F-59000 Lille, France; 37 Institute of Psychiatry and Neuroscience of Paris, University of Paris, INSERM U1266, Paris, France; 38 GHU Paris Psychiatrie et Neurosciences, CMME, Hôpital Sainte-Anne, Paris, France

**Keywords:** Confusional state, COVID-19, mental health, psychiatry

## Abstract

**Background:**

Patients with psychiatric disorders are exposed to high risk of COVID-19 and increased mortality. In this study, we set out to assess the clinical features and outcomes of patients with current psychiatric disorders exposed to COVID-19.

**Methods:**

This multi-center prospective study was conducted in 22 psychiatric wards dedicated to COVID-19 inpatients between 28 February and 30 May 2020. The main outcomes were the number of patients transferred to somatic care units, the number of deaths, and the number of patients developing a confusional state. The risk factors of confusional state and transfer to somatic care units were assessed by a multivariate logistic model. The risk of death was analyzed by a univariate analysis.

**Results:**

In total, 350 patients were included in the study. Overall, 24 (7%) were transferred to medicine units, 7 (2%) died, and 51 (15%) patients presented a confusional state. Severe respiratory symptoms predicted the transfer to a medicine unit [odds ratio (OR) 17.1; confidence interval (CI) 4.9–59.3]. Older age, an organic mental disorder, a confusional state, and severe respiratory symptoms predicted mortality in univariate analysis. Age >55 (OR 4.9; CI 2.1–11.4), an affective disorder (OR 4.1; CI 1.6–10.9), and severe respiratory symptoms (OR 4.6; CI 2.2–9.7) predicted a higher risk, whereas smoking (OR 0.3; CI 0.1–0.9) predicted a lower risk of a confusional state.

**Conclusion:**

COVID-19 patients with severe psychiatric disorders have multiple somatic comorbidities and have a risk of developing a confusional state. These data underline the need for extreme caution given the risks of COVID-19 in patients hospitalized for psychiatric disorders.

## Introduction

In March 2020, France became one of the first three European countries, after Italy and Spain, to be severely affected by COVID-19, the disease caused by severe acute respiratory syndrome coronavirus 2 (SARS-CoV-2). On 29 May 2020, 100 841 cases of COVID-19-related hospitalization were reported in France, of which 3960 cases concerned intensive care units. At the same time, 28 530 COVID-19-related deaths were reported, with about 36% of deaths in nursing homes or retirement homes (Salje et al., 2020).

Among the general population, several socio-demographic and clinical variables were associated with a high risk of hospital admission and complications in COVID-19 patients. Of these, older age, male sex, smoking, obesity, cardiovascular (CV), and respiratory disease were among the most frequently reported (Docherty et al., 2020; Petrilli et al., 2020; Richardson et al., 2020). Significantly, populations with mental illnesses are particularly concerned by some of these risk factors, since they show higher rates of respiratory disease, CV disease, tobacco use and metabolic syndrome (Correll et al., 2017; Gandré & Coldefy, 2020; Godin et al., 2015, 2019, 2014; Joukamaa et al., 2001; Laursen, Nordentoft, & Mortensen, 2014). Subjects diagnosed with a mental illness have a life expectancy 15–20 years shorter compared to the general population (Laursen et al., 2014). Wang, Xu, and Volkow (2020) recently conducted a study on electronic health records showing that patients with a recent diagnosis of mental disorder had a higher risk for COVID-19 infection than patients with no diagnosis of mental disorder, and an increased risk of death during COVID-19 infection. In similar vein, Li, Li, Fortunati, and Krystal (2020) conducted a cohort study based on medical records and also showed high mortality among patients hospitalized with COVID-19 with a psychiatric disorder compared to those with no psychiatric disorder. In a population-based study in patients hospitalized for COVID-19, Fond et al. (2020) showed that 60–80 years old patients with a previous diagnosis of schizophrenia had an increased mortality but a decreased intensive care unit admission rate. Moreover, it is worth noting that COVID-19 may be associated with confusional syndrome, which can be initially confounded with psychiatric symptoms (Varatharaj et al., 2020). Despite all this information, clinical description of psychiatric patients with a COVID-19 infection and of the risk factors associated with somatic aggravation or mortality is still missing.

Given the risks associated with COVID-19 infection and the frequency of high-risk comorbidities in psychiatric patients, specific COVID/PSY wards were created in French psychiatric hospitals (Chevance et al., 2020). At the beginning of the pandemics, this creation was also motivated by the risk of an overwhelming of medical wards or intensive care units. Patients admitted in these units had acute severe pre-existing psychiatric conditions that could not be treated outside the psychiatric wards and had symptoms of COVID-19 or were positive at SARS-CoV-2 testing. In these units, patients were treated by dedicated psychiatric and medical staff trained to provide treatment for the disease. In cases of COVID-19 symptom aggravation, patients were transferred to infectious care, pneumology or intensive care departments in general hospitals. Such COVID wards have also been opened in other countries such as Italy (Percudani, Corradin, Moreno, Indelicato, & Vita, 2020) or UK (Knowles et al., 2020). In the case of France, the opening of these units was promoted at the national level (Bocher, Jansen, Gayet, Gorwood, & Laprévote, 2020). Most of these units were created by conversion of existing psychiatric units. They included volunteer care teams from their hospital staff. Each COVID unit included one to two general practitioners and one to three psychiatrists. They had an average of 13 beds.

In this observational study, we set out to assess the clinical features of patients hospitalized in COVID/PSY wards in France and the risk factors associated with their clinical aggravation and mortality.

## Methods

### Study design

We conducted a multicenter prospective observational study of patients hospitalized in COVID/PSY wards at 22 psychiatric hospitals. Eight of these were public psychiatric hospitals, seven were university general hospitals containing psychiatric wards, six were public general hospitals containing psychiatric wards, and one was a private psychiatric hospital. The organization of all these COVID/PSY wards was based on the recommendations of the French Ministry of Health and Solidarities, i.e. specifically dedicated to patients requiring a psychiatric hospitalization with clinical suspicion of COVID-19 or positive for SARS-CoV-2 (Bocher et al., 2020; Chevance et al., 2020). These units had a dedicated medical and psychiatric staff. A total of 89 COVID/PSY wards were identified in France (Bocher et al., 2020). The 22 hospitals included in this study responded to a call for volunteers between 19 March 2020 and 10 April 2020 (24.7% of the COVID/PSY wards). They are distributed throughout the whole metropolitan French territory (Agen, Bayonne, Besançon, Bourges, Brumath, Cadillac, Caen, Créteil, Le Puy en Velay, Limoges, Lyon, Metz, Montpellier, Nancy, Neuilly sur Marne, Paris, Rennes, Saint Etienne, Saint Mandé, and Toulouse). This sample includes the units dedicated to the most affected French areas during the first peak of the COVID-19 pandemic, which included most of the patients (Brumath, Créteil, Lyon, Metz, Nancy, Neuilly sur Marne, and Paris). Due to the clinical constraints of the pandemic, we identified the variables that were essential to determine the socio-demographic status, the clinical and biological COVID-19 status, and the risk factors of somatic aggravation.

The authors assert that all procedures contributing to this work comply with the ethical standards of the relevant national and institutional committees on human experimentation and with the Helsinki Declaration of 1975, as revised in 2008. In accordance with French bioethics laws, it was classified as a non-interventional study, since it concerned anonymized clinical data that are routinely compiled in medical records. Compliance with French regulations on personal data collection was supervised by the personal data referent of the Centre Psychothérapique de Nancy. Patients, and their legal guardian when appropriate, were informed in writing, in agreement with national regulations for observational studies.

### Participants

We collected data from patients hospitalized in COVID/PSY wards between 28 February and 30 May 2020. This timeframe was determined because it corresponds to the opening dates of most COVID/PSY units during the first COVID-19 peak. After this first peak, many units were suspended and could be reactivated in case of a new peak or outbreak in the hospital. All patients with a psychiatric disorder requiring hospitalization and who presented a clinical diagnosis of COVID-19 were eligible for admission in COVID/PSY units and for inclusion in this study. At the beginning of the pandemic, reverse transcription-polymerase chain reaction (RT-PCR) testing were not systematically available in France and thus cases of COVID-19 were clinically defined according to the ICD-10 criteria, and classified in three clinical statuses: patients with an acute respiratory illness; patients with moderate clinical symptoms compatible with COVID-19 infection; and patients with asymptomatic or moderate clinical symptoms (contact cases, patients at risk/exposed to COVID-19). Severe respiratory illness was defined as the occurrence of cough (dry or wet), shortness of breath or hemoptysis, associated or not with other signs of infection. Three biological COVID statuses were possible: positive RT-PCR test, negative test or non-tested. Non-tested status was justified by the difficulties of access to biological tests at the beginning of the pandemic or of convincing patients to accept a nasopharyngeal swab. Evocative chest computerized tomography scans were also occasionally preferred to RT-PCR at the beginning of the pandemics since they had a comparable diagnostic performance (He et al., 2020).

### Description of collected data

The data were identified and collected prospectively in each center and were sent every week to the principal investigator via a secure encrypted data system. We permitted retrospective notification of cases occurring before the data collection system became available (19 March). The socio-demographic data included, age, sex, and the number of days of hospitalization in the COVID/PSY ward. The clinical COVID-19 status was based on ICD-10 and classified into three clinical statuses. The biological COVID-19 status was based on RT-PCR testing as mentioned above. The other clinical factors included tobacco smoking status (tobacco smoker/not smoker at admission), body mass index at admission (BMI), principal psychiatric diagnosis at admission according to ICD-10, non-respiratory clinical symptoms of COVID-19 (anosmia, digestive symptoms) at admission or during the hospitalization, and medical comorbidities defined by their presence at admission or in patient history. Psychiatric disorders with small population sizes in our sample were secondly grouped to facilitate the analysis (for instance, bipolar disorders were grouped with major depressive disorders and into affective disorders group).

### Outcomes

Our first outcome was the number of patients transferred to somatic care units (pneumology, infectious care, or intensive care departments). The recommendations for COVID/PSY wards specified that they should receive patients with stabilized respiratory function, and that any aggravation should be transferred to critical care (Ministère des Solidarités et de la Santé, 2020). Therefore, we considered any transfer to somatic care unit as a potential somatic aggravation. We also assessed the number of deaths during or immediately after the course of COVID/PSY hospitalization. We finally assessed the number of patients presenting a confusional state at admission or during follow-up. The definition of confusional state/delirium was based on the ICD-10 description: ‘an etiologically nonspecific organic cerebral syndrome characterized by concurrent disturbances of consciousness and attention, perception, thinking, memory, psychomotor behaviour, emotion, and the sleep-wake schedule’.

### Statistical analysis

At baseline, continuous variables data were described as median, range [interquartile range (IQR)], and categorical variables as frequencies (percentages). Variables were compared using *t* test or χ^2^ test, as required.

We assessed the risk factors associated with the occurrence of a confusional state, and those associated with the transfer to somatic care units, by using a multivariate logistic regression model. We adjusted for significantly different variables (*p* < 0.05) at baseline/admission. The following variables were tested in the univariate analysis: age, sex, tobacco smoking status (actual smoker/not smoker), BMI, principal psychiatric diagnosis, clinical COVID status (severe *v.* moderate/absent respiratory symptoms), other symptoms at admission (anosmia, digestive symptoms), biological COVID-19 status (PCR-positive *v.* PCR-negative/not tested), days of hospitalization in the clinical COVID units, and comorbidities. The following medical comorbidities were tested: hypertension, diabetes, asthma, chronic obstructive pulmonary disease (COPD), coronary artery disease (CAD), heart failure (HF), and other chronic diseases. Simulations were performed by adjusting for variables that were correlated with the outcome up to *p* = 0.2 and the results were similar.

Assumptions of log-linearity were checked as follows. We categorized the continuous variables into quintiles, created dummy variables per quintile of each variable, and fitted models with these dummy variables. We then plotted the beta-estimators against the mean values of the quintiles. Based on the log-linear criteria we reclassified the continuous variable age (age >55 years).

Due to the small number of death events, we could not perform a multivariate logistic analysis to assess the multivariate predictors of mortality, and thus we analyzed only the univariate predictors of this outcome.

All analyses were performed using SPSS version 24. The results were estimated as odds ratios (ORs) with 95% confidence intervals (CIs). The two-tailed significance level was set-up at *p* < 0.05.

## Results

### Patient inclusion

**Fig. 1.**
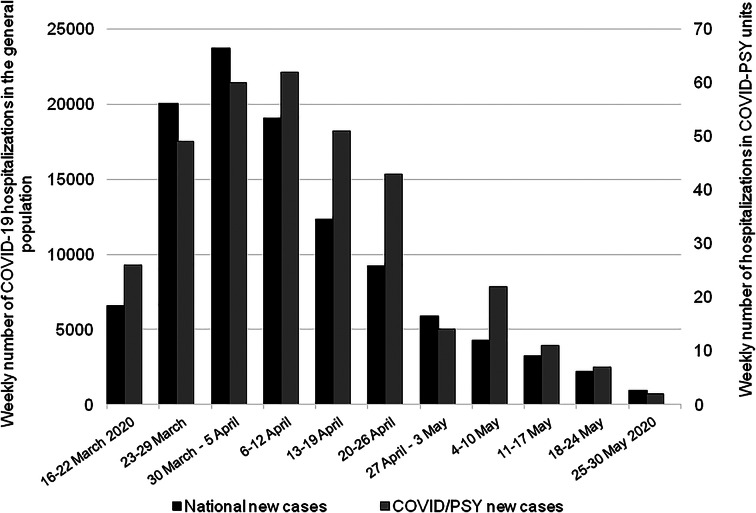
Distribution of weekly number of hospitalizations in COVID/PSY wards (gray) and of weekly number of COVID-19 hospitalizations in the general population (black). To facilitate the comparison of the evolution of hospitalization over time, it should be noted that two different scales are used in the general population and in COVID/PSY units.

In total, 350 patients were included in the study. Figure 1 shows the weekly number of patients included in our study and the weekly number of COVID-19 hospitalizations in the French general population between 28 February and 30 May 2020 provided by the Santé Publique France database (Santé Publique France, s. d.). In COVID/PSY wards, the number of cases began to increase as of week 12 (16–22 March) and the peak of the inclusion curve was reached during week 15 (6–12 April). In the general population, the peak of the inclusion curve was reached during week 14 (30 March–5 April). The number of patients included in COVID/PSY units appears to have followed by 1 week the peak of COVID-19 observed in the French general population. However, the dynamic of the peak appears to be slightly different with a slower decline of the cases during April and May 2020 in COVID/PSY units.

### Patient characteristics

The main patient characteristics are presented in Table 1. The majority of patients (42%) were hospitalized for schizophrenia or psychotic disorder, or for an affective disorder (23%), including depression or bipolar disorder. The median (IQR) age of patients was 50 (36–63) years and 55% were male. In total, 39% of patients were tobacco smokers, and the median BMI was 25 (21–28). The patients presented several CV, respiratory and metabolic comorbidities, such as hypertension (22%), diabetes (12%), COPD (9%), and asthma (7%). In addition, the patients presented other chronic severe diseases, including neurological diseases (7%), chronic infectious diseases (3%), autoimmune diseases (2%), or cancer (2%). Overall, the patients were hospitalized in the COVID/PSY wards for a median of 9 (4–14) days.

**Table 1.** Socio-demographic characteristics and clinical features of patients hospitalized in COVID/PSY units

Variable	Total patients (*N* = 350)
Socio-demographic data	
Age, years, median (range; IQR)	50 (17–97; 36–63)
Age >55 years, *n* (%)	139 (40%)
Sex, men, *n* (%)	194 (55%)
Principal psychiatric diagnosis, *n* (%)	
Schizophrenia/psychosis	147 (42%)
Affective disorder (depression/bipolar disorder)	81 (23%)
Personality disorder	27 (8%)
Psychological development disorder	25 (7%)
Anxious disorder	17 (5%)
Drug use-related disorder	11 (3%)
Eating disorder	7 (2%)
Intellectual disability	12 (3%)
Organic mental disorder	8 (2%)
Others	13 (4%)
Comorbidities, lifestyle, *n* (%)	
Smoking^a^	138 (39%)
BMI^b^, median (range; IQR)	25 (12–50; 21–28)
Diabetes	41 (12%)
Asthma	23 (7%)
COPD	30 (9%)
Hypertension	76 (22%)
CAD	6 (2%)
HF	4 (1%)
Neurological disease	26 (7%)
Chronic infectious disease	10 (3%)
Autoimmune disease	8 (2%)
Cancer	11 (3%)
Clinical COVID-19 symptoms, *n* (%)	
Acute severe respiratory symptoms	111 (32%)
Moderate respiratory symptoms	178 (51%)
Asymptomatic/moderate symptoms (contact cases)	60 (17%)
Other symptoms at admission, *n* (%)	
Digestive symptoms	61 (17%)
Anosmia	21 (6%)
Confusional state, *n* (%)	51 (15%)
Biological COVID-19 status (PCR test), *n* (%)	
Positive	176 (50%)
Negative	154 (44%)
Non-tested	20 (6%)
Days hospitalization, median (range; IQR)	9 (1–46; 4–14)
Transfer to intensive care/ somatic units, *n* (%)	24 (7%)
Death, *n* (%)	7 (2%)

a Data available for 332 patients.

b Data available for 306 patients.

Regarding clinical COVID-19 symptoms, 32% of the patients presented acute severe respiratory symptoms, while 51% presented moderate respiratory symptoms and 17% were asymptomatic or moderate symptomatic contact cases. When grouping the two later classes, 68% of patients presented moderate symptoms or were asymptomatic. In addition, 17% of patients presented digestive symptoms and 6% presented anosmia. Overall, 50% of patients were positive at RT-PCR testing, 44% were negative, and 6% could not be tested.

### Outcomes

During their hospitalization in COVID/PSY units, 24 (7%) patients were transferred to somatic care units and 7 (2%) patients died. At the same time, 51 (15%) patients presented a confusional state at admission or during follow-up.

#### Transfer to somatic care units

Table 2 presents the variables associated with the transfer to somatic care units during the study follow-up. In univariate logistic analysis, patients with COPD had three times higher risk of transfer (OR 3.2; CI 1.1–9.2; *p* = 0.03), whereas those with severe respiratory symptoms had 18 times higher risk compared to moderate/asymptomatic patients (OR 18.3; CI 5.3–62.8; *p* < 0.001). In multivariate analysis, only the severe respiratory symptoms remained highly predictive of the transfer to somatic care units (OR 17.1; CI 4.9–59.3; *p* < 0.001).

**Table 2.** Risk factors associated with the transfer in intensive care/other medicine units

Variable/outcome	Univariate analysis		Multivariate analysis	
	OR (95% CI)	*p* value	OR (95% CI)	*p* value
COPD	3.2 (1.1–9.2)	0.03	1.8 (0.6–5.7)	0.3
Severe respiratory symptoms *v.* moderate/asymptomatic symptoms	18.3 (5.3–62.8)	<0.001	17.1 (4.9–59.3)	**<0.001**

COPD, chronic obstructive pulmonary disease.

#### Death

Table 3 shows the variables associated with the risk of death in univariate logistic analysis. Primarily, as expected, age was highly predictive of death, with a median age of deceased patients of 72 (54–80) years, as opposed to a median age of 50 (35–62) years for survivors (OR 1.1; CI 1.02–1.1; *p* = 0.009). Furthermore, the occurrence of a confusional state increased the risk of death eightfold (OR 8.4; CI 1.8–38.7; *p* = 0.001) and the occurrence of severe respiratory symptoms increased the risk almost sixfold (OR 5.6; CI 1.1–29.2; *p* = 0.02). Finally, the strongest predictor of death was a diagnosis of an organic mental disorder, such as dementia (OR 22.5; CI 3.6–139.7; *p* = 0.001).

**Table 3.** Univariate risk predictors of mortality

	Deceased patients (*N* = 7)	Survivors (*N* = 343)	Univariate logistic analysis	
			OR (95% CI)	*p* value
Age, years, median (IQR)	72 (54–80)	50 (35–62)	1.1	**0.009**
Organic mental disorders	2 (29%)	6 (2%)	22.5 (3.6–139.7)	**0.001**
Confusional state	4 (57%)	47 (14%)	8.4 (1.8–38.7)	**0.001**
Severe respiratory symptoms *v.* moderate/asymptomatic symptoms	5 (71%)	106 (31%)	5.6 (1.1–29.2)	**0.02**

It is of note that a principal diagnosis of intellectual disability was also associated with a trend toward a higher risk of death, but the variable did not reach statistical significance, probably due to the small number of death events in our study (OR 5.0; CI 0.6–45.4; *p* = 0.1).

#### Confusional state

Table 4 presents the variables associated with the occurrence of a confusional state in univariate and multivariate logistic analyses. In univariate analysis, patients >55 years old had an eightfold higher risk of confusional state compared to younger patients (OR 8.4; CI 4.0–17.5; *p* < 0.001). Patients with COPD and those with hypertension had also about two and three times higher risk, whereas patients with acute respiratory symptoms were at almost five times higher risk than the group of moderate symptomatic/asymptomatic patients (OR 4.7; CI 2.5–8.7; *p* < 0.001). Interestingly, patients with affective disorders (depression or bipolar disorder) had almost four times higher risk of developing a confusional state (OR 3.7; CI 1.7–8.0; *p* < 0.001). In contrast, smokers had a lower risk of developing a confusional state (OR 0.2; CI 0.1–0.5; *p* < 0.001).

**Table 4.** Risk factors associated with the occurrence of a confusional state

Variable/outcome	Univariate analysis		Multivariate analysis	
	OR (95% CI)	*p* value	OR (95% CI)	*p* value
Age >55 years	8.4 (4.0–17.5)	<0.001	4.9 (2.1–11.4)	**<0.001**
Hypertension	3.4 (1.8–6.4)	<0.001	1.5 (0.7–3.3)	0.3
Affective disorder (depression/bipolar disorder)	3.7 (1.7–8.0)	0.001	4.1 (1.6–10.9)	**0.004**
COPD	2.3 (0.9–5.6)	0.05	1.7 (0.5–5.1)	0.4
Severe respiratory symptoms *v.* moderate/asymptomatic symptoms	4.7 (2.5–8.7)	<0.001	4.6 (2.2–9.7)	**<0.001**
Smoking	0.2 (0.1–0.5)	<0.001	0.3 (0.1–0.9)	**0.02**

COPD, chronic obstructive pulmonary disease.

In multivariate analysis, four clinical variables remained associated with the risk of developing a confusional state. Older age (age >55 years) (OR 4.9; CI 2.1–11.4; *p* < 0.001), severe respiratory symptoms (OR 4.6; CI 2.2–9.7; *p* < 0.001), and a diagnosis of affective disorder (OR 4.1; CI 1.6–10.9; *p* = 0.004) predicted a higher risk, while smoking (OR 0.3; CI 0.1–0.9; *p* = 0.02) predicted a lower risk of a confusional state.

## Discussion

In this multicenter prospective study, we aimed to assess the clinical features and the risk factors associated with clinical aggravation and mortality in 350 patients hospitalized in COVID/PSY units in France during the first pandemic wave of COVID-19. During their hospitalization in COVID/PSY units, 7% of patients were transferred to a somatic care unit. Severe respiratory symptoms were the only variable associated with the transfer to a somatic care unit. Moreover, 2% of patients hospitalized in COVID/PSY units died. Older age, the presence of a confusional state, severe respiratory symptoms, and a diagnosis of organic mental disorder were predictive of mortality in univariate analysis. Finally, 15% of patients developed a confusional state at admission or during follow-up. Age >50, a diagnosis of depression/bipolar disorder, and the occurrence of severe respiratory symptoms were associated with a higher risk, whereas smoking was predictive of a lower risk of this syndrome.

To our knowledge, this is the only study presenting the clinical features and the risk factors of somatic aggravation in patients hospitalized for psychiatric disorders and exposed to COVID-19 infection. Two previous cohort studies based on medical records respectively showed increased risks of COVID-19 infection and mortality in patients with a psychiatric diagnosis (Wang et al., 2020) and a higher risk of mortality in patients hospitalized for COVID-19 when they had a diagnosis of psychiatric disorder (Li et al., 2020). Interestingly, the mortality rate of 2% found in COVID/PSY wards was markedly lower than the mortality rate of 8.5% measured by Wang et al. (2020), although this comparison should be made with caution, as it involves two different countries and health systems. On the basis of the French national hospital database, Fond et al. (2020) showed a decreased access to intensive care in 60–80 years old patients with schizophrenia. In our study, all the patients who died had a prior access to intensive care. Moreover, no denial of access to intensive care was noted as soon as the need for transfer was decided in COVID/PSY wards. This could be an indication of the effectiveness of support by COVID/PSY units when a transfer was required.

The daily number of patients included in COVID/PSY units appears to peak 1 week after the general population, and a slower decline of the epidemic curve was observed during April and May 2020 in COVID/PSY units. This point suggests that a great prudence should be applied in populations suffering from psychiatric diseases during the descending phases of the COVID-19 pandemic.

Despite their relatively young age, the patients in our study presented a broad range of comorbidities, including hypertension (20%), diabetes (12%), and neurological disorders (7%), but also respiratory disorders, which are frequent among patients with psychiatric disorders, especially those with schizophrenia/psychosis. The high percentage of patients with COPD (9%) and asthma (8%) is noteworthy and most probably related to the percentage of smokers in this population (39%). Although the COPD was a predictor of clinical aggravation (transfer to a medicine unit) in univariate analysis, it was not however retained as a significant variable in multivariate analysis. There are probably other clinical or biological factors independent of COPD and non-included in this study that may explain the occurrence of clinical aggravation in this population.

One interesting finding of our study is the occurrence of a confusional state in a relatively high percentage of patients (15%). Significantly, the occurrence of a confusional state is also associated with a high risk of death. Neurological and central nervous system manifestations of COVID-19 have been reported in several studies (Ahmad & Rathore, 2020; Asadi-Pooya & Simani, 2020; Chen et al., 2020; Mao et al., 2020; Wu et al., 2020). In a retrospective study of 99 cases of COVID-19 with pneumonia, nine (8%) patients presented the symptoms of confusion (Chen et al., 2020). In a retrospective case series study of 214 hospitalized patients, 78 (36%) patients had neurological manifestations, such as acute cerebrovascular disease (3%), impaired consciousness (7%), and skeletal muscle injury (11%). Significantly, patients with severe infection were more likely to develop neurological manifestations, including impaired consciousness, i.e. 15% in severe *v.* 2% in non-severe patients (Mao et al., 2020). Several mechanisms of neurological manifestations have been presented, including the direct neurotropism of the coronavirus (direct injury), the hypoxic mechanism via the damage of the pulmonary tissue (indirect hypoxic neuronal injury), or the immune-mediated (indirect) neuronal injury mechanism, mainly due to the increased levels of inflammatory cytokines and activation of T-lymphocytes (Ahmad & Rathore, 2020; Asadi-Pooya & Simani, 2020; Wu et al., 2020). Patients with severe psychiatric disorders may be at particular risk of this syndrome, due to the mechanisms inherent to their pathology and its treatment.

Although older age and severe infectious disease are known to be the predictors of a confusional syndrome, it is of clinical interest that in our study a diagnosis of major depressive disorder or bipolar disorder was also predictive of confusional syndrome. SARS-CoV-2 can activate deregulated host immune responses via elevation of cytokines such as interleukin-1*β* (IL-1*β*), interleukin-6 (IL-6), IP-10, tumor necrosis factor *α* (TNF-*α*), interferon-*γ* or macrophage inflammatory protein (MIP) 1*α* and 1*β* (Fajgenbaum & June, 2020). This response may contribute to the severity of COVID-19 (Coomes & Haghbayan, 2020). On the contrary, the elevated serum levels of IL-1*β*, IL-6, and TNF-*α* have been repeatedly observed in major depressive disorder (Miller & Raison, 2016) and in bipolar disorder (Sayana et al., 2017). Several authors recently suggested an interaction between this chronic inflammatory state in affective disorders and the cytokine storm triggered by COVID-19 (Steardo, Steardo, & Verkhratsky, 2020; Tamouza, Krishnamoorthy, & Leboyer, 2021). This interaction could lead to confusional syndrome, the severity of which is clearly associated with high levels of cytokines under various medical conditions (Khan et al., 2020).

It is also of clinical interest that smoking was predictive of a lower risk of confusional state in our population. The prevalence of smokers is surprisingly low in this sample compared to other French studies: Mallet et al. (2017) found 53.7% smokers in outpatients with schizophrenia, whereas Poirier et al. (2002) found 58.9% smokers in a sample of in- and outpatients with mental disorders. It should be noted that several studies have pointed to the under-representation of tobacco smokers among those hospitalized for COVID-19 (González-Rubio et al., 2020; Simons, Shahab, Brown, & Perski, 2020). These findings have to be interpreted with caution (Usman et al., 2020) as it is well known that tobacco smoking is a major risk factor for severe respiratory illness, and smokers in general experience higher rates of influenza and bacterial pneumonia (Eapen, Sharma, Moodley, Hansbro, & Sohal, 2019; Tuder & Yun, 2008). However, Farsalinos et al. recently showed *in silico* that there was an interaction between SARS-CoV-2 and nicotinic receptors, which could implicate a protective role of nicotine against SARS-CoV-2 (Farsalinos et al., 2020). Beside these findings, several studies reported that nicotine inhibits the production of pro-inflammatory cytokines (TNF-*α*, IL-1, and IL-6) (Ulloa, 2005; Wang et al., 2003), and these effects have been shown to protect against cytokine-mediated diseases which can lead to organ damage as from a ‘cytokine-storm’, the main culprit of COVID-19 (Conti et al., 2020). Undoubtedly, more research is needed to examine the interaction between tobacco smoking and the virulence of COVID-19.

Our study has several strengths. To our knowledge, this is the first large multicenter prospective observational study conducted in psychiatric hospitals during the COVID-19 pandemic. Patients were recruited and followed up in 22 hospitals, with dedicated COVID/PSY units. We also assessed an important number of socio-demographic and clinical characteristics, as well as relevant clinical outcomes for this specific population.

Our study also has its own limitations. Overall, only 50% of the included patients were positive at SARS-CoV-2 testing. This proportion is comparable to the 47% of positive patients found in the observational study of Livingston et al. (2020) in patients with dementia during a similar timeframe. RT-PCR testing was performed at the beginning of the hospitalization in COVID/PSY wards or as soon as patient's behavior could allow it. At the beginning of the pandemic RT-PCR testing was scarcely available and its reliability has been challenged (Axell-House et al., 2020). The high percentage (44%) of patients negative at RT-PCR testing could be explained by the flaws in the testing methodology at the beginning of the pandemic, which may have resulted in a number of false-negative cases. Therefore, patients have been admitted in COVID/PSY units with a negative RT-PCR test but an evocative chest scan, in compliance with studies that proposed chest scan as an alternative diagnostic confirmation for COVID-19 at the beginning of the pandemic (He et al., 2020). Moreover, this study did not identify psychotropic treatments during the COVID-19 episode. This is a limitation because such treatments could have interfered with the course of COVID-19 or the occurrence of confusional syndrome. This choice was made when designing this study because of the very limited time to train the teams and implement the study. Some of the teams in this study were unfamiliar with the research procedures and we chose to restrict the data to simple nominal data. Another limitation was the small number of death events and therefore we analyzed only the univariate predictors of mortality. This element should be interpreted cautiously. Future studies with a larger recruitment could help consolidate these findings.

During the first peak of the COVID-19 pandemic, the French specific COVID/PSY wards admitted patients with psychiatric disorders and with COVID-19. These patients had a broad range of comorbidities, and those developing severe respiratory symptoms were at risk of transfer in other medicine services. In addition, these patients were at high risk of a confusional state, especially older patients with an affective disorder and severe symptoms. Finally, older patients with an organic mental disorder, a confusional state, and severe respiratory symptoms may have a higher risk of death. Despite these risks, patients hospitalized in COVID/PSY wards had a limited mortality rate, and their access to intensive care was facilitated when required. Such COVID/PSY wards should be available for psychiatric populations according to the evolution of the pandemic, in order to protect patients with psychiatric disorders and to limit the spread of SARS-CoV-2.
